# Editorial: Highly Mutable Animal RNA Viruses: Adaptation and Evolution

**DOI:** 10.3389/fmicb.2017.01785

**Published:** 2017-09-15

**Authors:** Masako Nomaguchi, Akio Adachi

**Affiliations:** ^1^Department of Microbiology, Tokushima University Graduate School of Medical Science Tokushima, Tokushima, Japan; ^2^Department of Microbiology, Kansai Medical University Hirakata, Osaka, Japan

**Keywords:** animal viruses, RNA viruses, mutation, adaptation, evolution

One of the most conspicuous and fundamental characteristics of animal RNA viruses is their high mutability under various circumstances. This property is a major cause for viral adaptation to changing environments, and also for medical problems/issues associated with viruses. In this Research Topic, numerous researchers have described and discussed the latest and/or momentous results on animal RNA viruses as original (27 papers), methods (1 paper), review (3 papers), mini-review (3 papers), perspective (1 paper), and general commentary (1 paper) articles. Given that each virus has its own strategy for its replication, transmission, and survival, we are justified to categorize individual articles into 8 groups by the target virus in question: (I) *Retroviridae-lentivirinae* (human and simian immunodeficiency viruses, 10 articles), (II) *Orthomyxoviridae* (influenza virus, 10 articles), (III) *Paramyxoviridae* (avian Newcastle disease virus, human metapneumovirus, and human respiratory syncytial virus, 5 articles), (IV) *Flaviviridae* (classical swine virus, hepatitis C virus, and Zika virus, 4 articles), (V) *Rhabdoviridae* (rabies virus, 2 articles), (VI) *Caliciviridae* (norovirus, 2 articles), (VII) *Picornaviridae* (enterovirus species C, and rhinovirus, 2 articles), and (VIII) *Picobirnaviridae* (picobirnavirus, 1 article).

In group (I) articles, researchers have summarized, reviewed, discussed and/or studied the (i) multiple ways of human and simian immunodeficiency viruses (HIV/SIVs) for modulation of viral/cellular gene expression, (ii) diversification (quasispecies) of Env caused by drug/immune pressure, (iii) anti-viral cellular factors against HIV/SIVs that greatly affect their biological properties, and (iv) the potential link between accessory Vpx/Vpr proteins and phylogenetic clusters of HIV/SIVs. Heusinger and Kirchhoff have described the viral/molecular mechanisms by which HIV/SIVs regulate anti-viral as well as viral gene expression in a viral replication cycle. Viral Tat protein controls the transcriptional activity of HIV/SIVs, and thus highly influences viral replication rates. Indeed, it has been demonstrated that naturally occurring Tat variations affect HIV type 1 (HIV-1) replication stage *in vivo* (Kamori and Ueno; Ronsard et al.). Also, Langer and Sauter have described various HIV-1 proteins including non-canonical ones expressed from its genome. As unique properties of HIV-1, Harada and Yoshimura, and Fujita have summarized and discussed the works on Env and on anti-viral factors in macrophages, respectively. Okada and Iwatani have described and discussed the molecular action mechanism of APOBEC3G, a major restriction factor against HIV-1. Finally, it has been reasonably postulated that adaptive mutations/variations may have contributed to the diversifications of HIV/SIVs (Sakai et al.; Sakai et al.). Miyazaki et al. has described distinct *in vitro* properties of HIV-1 and HIV-2 capsid proteins.

Needless to say, the emergence of new influenza viruses (IFVs) that are highly pathogenic to humans represents a major medical issue of great urgency. In group (II) articles, researchers have studied IFVs (mainly of the avian origin) with a special reference to their biological properties (pathogenicity for host individuals, host responses, intra- and inter-species transmission, genetic re-assortment, etc.). The variable nature of the influenza virus has been well documented, and solid anti-viral strategies based on the reported results are immediately required. The pathogenicity of H5N1, H5N6, H7N1, and H9N2 IFVs have been studied, and the results obtained have been discussed about the future direction (Jiao et al.; Gao et al.; Jin et al.; Liu et al.). While Meng et al. have studied the production of cytokines/chemokines in infected cells, Gao et al. have analyzed the host immune-related response and viral transmission efficiency in chickens. Karnbunchob et al. have focused on tracking viral avian-swine transmission, and found that most amino acid substitutions in the polymerase genes were acquired after interspecies transmission. Studies of Song et al., Yuan et al., and Zhao et al. have revealed that new viruses such as H1N2, H5N6, and H7N2, respectively, are generated by genomic reassortments. Finally, Yokoyama et al. have demonstrated, by molecular dynamics simulations, the structural basis for adaptive mutations of H3N2 IFV, a major cause for seasonal influenza in humans.

In group (III) articles, researchers have performed comprehensive genome analyses of avian or human paramyxoviruses coupled with pathogenicity studies. Satharasinghe et al. sequenced the full genome of a large number of Newcastle disease virus (NDV), showing the diversity of this virus in Malaysia. Different tissue tropisms/host ranges and pathogenicity-related host responses have been observed for various NDV strains in China (Kang et al.; Xu et al.). Saikusa et al. have systematically determined G genes of human metapneumovirus (HMPV) isolates in Japan, major causative viruses for acute respiratory diseases in humans, and have found a unique 180-nucleotide duplication in the corresponding sites of major epidemic HMPV strains. Zou et al. have determined numerous G genes of numerous respiratory syncytical virus (RSV) isolates in China, and have shown that a particular RSV genotype spreads rapidly and causes epidemics.

Flaviviruses are extraordinarily variable from biological and molecular biological points of view and are the topic of group (IV) articles. Hu et al. have determined E2 genes derived from numerous classical swine fever virus (CSFV) isolates from C-strain-vaccinated pigs to know the etiological reason for sporadic CSF outbreaks in the area. The results have shown that circulating CSFVs in those pigs mostly belong to a specific sub-genotype. Kim et al. have examined a retinoid-interferon-induced cell-mortality factor designated GRIM-19 for its effect on hepatitis C virus (HCV) replication, and suggested that GRIM-19 acts as an anti-viral host factor by reducing intracellular lipid accumulation. A virus designated Zika (ZIKV) has recently become the focus of public attention due to a remarkable number of microcephaly cases in Brazil. As for ZIKV, Saiz et al. have extensively reviewed and summarized the present knowledge, from basic information on biology and molecular biology, medical issues, and through to public health matters. Koide et al. have reported that the cynomolgus macaque can serve as an infection model for ZIKV.

In group (V) to (VIII) articles, rabies virus (RABV), noroviruses of murine (MuNoV) or human (NoV) origin, picornaviruses (enterovirus species C (EV-C) and human rhinovirus C (HRV-C)), and picobirnaviruses (PBVs) have been studied. Jamalkandi et al. have started the systems biology to identify a network of proteins significant in the RABV infection. The importance of RABV P protein for viral replication and pathogenicity has been shown by genetic manipulation as expected (Mei et al.). Kitamoto et al. have focused on the laboratory adaptation of MuNoV, and have suggested that the interplay between variants is necessary for the virus to better adapt for growth in cell culture. Sato et al. have performed an integrated analysis on NoV GII.4, a major cause of viral gastroenteritis in humans. They have examined the pandemic lineage for its molecular evolution by using viral full-length genome and VP1 sequences from a large number of samples collected in Japan. The results are important to understand the biology of NoV and to control NoV infection. Bessaud et al. have successfully developed a method to sequence rapidly the entire EV-C genome. It is useful to identify the type of EV-C strain and also to distinguish it from closely related viruses. Khaw et al. have determined the complete genome sequence of seven Malaysian HRV-C isolates, and found unique and conserved sequences relative to that of HRV-Cs from the other countries. Finally, Woo et al. have determined partial sequences of PBVs isolated from numerous animal species, and performed a phylogenetic analysis. PBVs have been recently discovered in a wide variety of mammals and birds. The results obtained have demonstrated a highly divergent nature of PBV.

As mentioned in the first part of this article, there are 36 papers in this Research Topic covering a wide variety of animal RNA viruses. Each work has its own scientific impact, and highlights virological significance and interest of the virus diversification. Animal RNA viruses readily mutate in response to certain biological and/or chemical stimuli rendered by the surrounding environments. To precisely know or understand the biological processes and the underlying molecular mechanisms remains a mission for virologists, requiring both applied as well as basic virology.

## Author contributions

MN and AA wrote the manuscript, and approved its submission.

### Conflict of interest statement

The authors declare that the research was conducted in the absence of any commercial or financial relationships that could be construed as a potential conflict of interest.

